# Comparing Different Registration and Visualization Methods for Navigated Common Femoral Arterial Access—A Phantom Model Study Using Mixed Reality

**DOI:** 10.3390/jimaging10040076

**Published:** 2024-03-25

**Authors:** Johannes Hatzl, Daniel Henning, Dittmar Böckler, Niklas Hartmann, Katrin Meisenbacher, Christian Uhl

**Affiliations:** 1 Department of Vascular and Endovascular Surgery, University Hospital Heidelberg, 69120 Heidelberg, Germany; 2 Department of Vascular Surgery, University Hospital RWTH Aachen, 52074 Aachen, Germany

**Keywords:** mixed reality, virtual reality, endovascular, intervention, vascular surgery, vascular access, interventional radiology

## Abstract

Mixed reality (MxR) enables the projection of virtual three-dimensional objects into the user’s field of view via a head-mounted display (HMD). This phantom model study investigated three different workflows for navigated common femoral arterial (CFA) access and compared it to a conventional sonography-guided technique as a control. A total of 160 punctures were performed by 10 operators (5 experts and 5 non-experts). A successful CFA puncture was defined as puncture at the mid-level of the femoral head with the needle tip at the central lumen line in a 0° coronary insertion angle and a 45° sagittal insertion angle. Positional errors were quantified using cone-beam computed tomography following each attempt. Mixed effect modeling revealed that the distance from the needle entry site to the mid-level of the femoral head is significantly shorter for navigated techniques than for the control group. This highlights that three-dimensional visualization could increase the safety of CFA access. However, the navigated workflows are infrastructurally complex with limited usability and are associated with relevant cost. While navigated techniques appear as a potentially beneficial adjunct for safe CFA access, future developments should aim to reduce workflow complexity, avoid optical tracking systems, and offer more pragmatic methods of registration and instrument tracking.

## 1. Introduction

Mixed reality (MxR) technology enables the projection of virtual three-dimensional objects into the user’s field of view through a head-mounted display (HMD). These virtual objects can be generated from cross-sectional imaging, such as computed tomography angiography (CTA), offering various potential applications in surgery and interventional radiology [1,2,3,4,5,6,7,8,9,10,11,12]. MxR-assisted intraoperative navigation has the potential to increase the safety for a variety of procedures, including common femoral arterial (CFA) access [13,14].

CFA access requires the exact percutaneous puncture of the common femoral artery at the mid-level of the femoral head on the anterior wall of the vessel. It is frequently performed worldwide in common procedures such as coronary angiography, peripheral vascular interventions, stroke therapy, and others. Complications related to CFA access can have serious consequences with high morbidity and mortality. Relative frequencies of complications are reported to be rather low. However, considering the absolute number of procedures performed, these complications still represent a relevant clinical finding in daily practice [15]. Furthermore, larger profile vascular access such as in thoracic endovascular aortic repair or fenestrated and branched aortic repair is associated with access site complications or closure device failures of up to 5–10% [16,17]. Known risk factors are insufficient puncture location, calcification, and target vessel morphology [18,19,20,21]. Achieving safe femoral arterial access requires a comprehensive visualization of the common femoral artery bifurcation, inguinal ligament, and calcifications. While sonography is a standard method for assisting CFA access, complications may still arise due to limited visualization in two simultaneous dimensions [15,22,23,24,25,26,27].

Reliable and clinically feasible registration of the physical and virtual patients as well as needle tracking are currently the main technical challenges of MxR-assisted navigation [28]. The standard method of paired-point registration involves using superficial fiducial markers on the patients’ skin. While its accuracy has been shown in different clinical contexts, this method faces challenges related to marker displacement and patient positioning, especially in soft tissue applications such as CFA access [29,30].

Alternative registration approaches use a sonography-assisted method utilizing a deep learning algorithm based on sonography swipe images. This method eliminates the need for superficial markers and potentially enhances the clinical applicability of MxR.

This study aims to compare the usability and positional accuracy of three different navigated workflows for CFA access techniques on a phantom model across diverse anatomies and with operators of varying experience levels. The goal is to compare these methods against the traditional sonography-guided technique as a control.

## 2. Materials and Methods

### 2.1. Phantom Model

**Figure 1:**
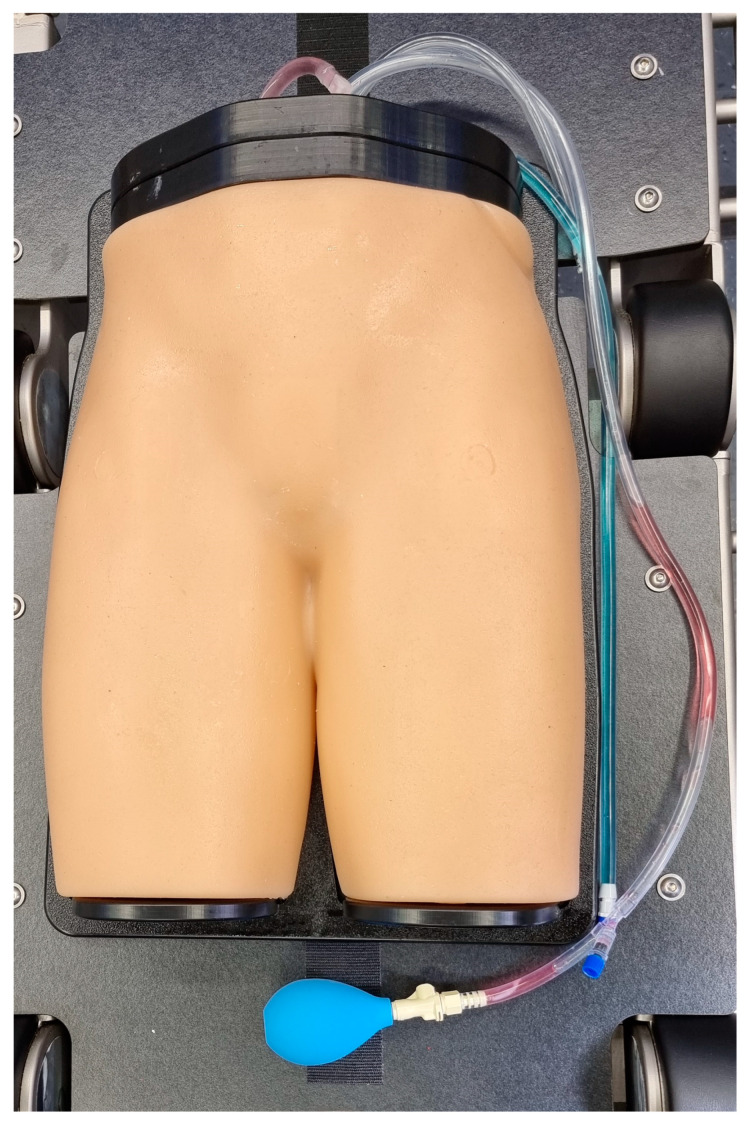
Gen II Femoral Vascular Access and Regional Anesthesia Ultrasound Training Model (CAE Healthcare, Mainz, Germany).

**Figure 2:**
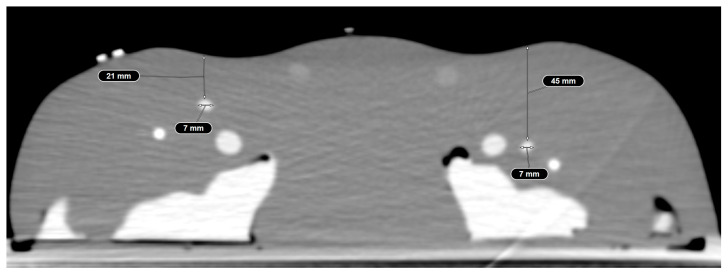
Computed tomography angiography of the phantom model displaying realistic tissue differentiation.

A Gen II Femoral Vascular Access and Regional Anesthesia Ultrasound Training Model (CAE Healthcare, Mainz, Germany) was used for the purposes of this study. The characteristics of the model have been previously described [13]. In short, it includes arterial and venous vasculature as well as femoral joints, inguinal ligaments, and the femoral nerve. The model’s soft tissue allows more than 1000 punctures per square centimeter with minimal residual signs of previous puncture attempts. It can be examined using sonography and computed tomography angiography producing live-like images with realistic tissue differentiation. A contrast CT scan of the model was performed using an AIRO 32 slice CT scanner (Brainlab AG, Munich, Germany). Punctures were performed using a 14 G Vasofix peripheral venous catheter needle (B. Braun SE, Melsungen, Germany) (10 cm in length). The target vessel (CFA) at the level of the planned trajectory is localized at a depth of 21 mm and has a diameter of 7 mm on the right side, and on the left side, it is localized at a depth of 45 mm and has a diameter of 7 mm. Therefore, the left side was defined as anatomically more challenging. The phantom model is displayed in Figure 1 and Figure 2.

### 2.2. Head-Mounted Display

**Figure 3:**
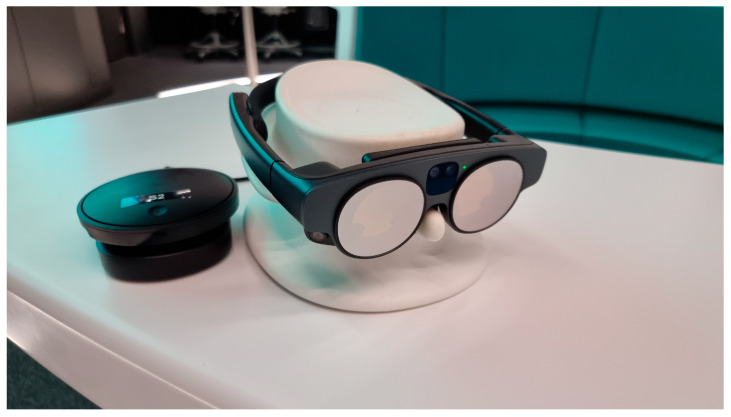
MagicLeap 2 (MagicLeap, Plantation, FL, USA).

The Magic Leap 2 (MagicLeap, Plantation, FL, USA) has been used as the HMD in this study. It was introduced to the market in September 2022 and has received IEC 60,601 certification. This allows it to be used in a clinical setting including operating rooms. The Magic Leap 2 is displayed in Figure 3.

### 2.3. Workflows of Navigated CFA Access Techniques

Each of the three navigated workflows consisted of two main steps: the registration of the phantom model with the virtual object and the subsequent visualization made available to the operator. The three navigated workflows were then compared to a conventional sonography-guided control.

With regard to registration, two different methods were used for the purposes of this study.

The first method of registration was paired-point registration. Following the acquisition of a computed tomography scan with five fiducial markers in place on the surface of the model, the phantom is then placed on the operating table. The optical tracking reference array is positioned next to the model and in the field of view of the optical tracking camera of the Curve^®^ navigation system (Brainlab AG, Munich, Germany). Subsequently, the radiopaque fiducial markers are defined as reference landmarks and acquired by the model using a tracked pointing device. The process of paired-point registration is demonstrated in Supplementary Video S1 and has previously been described for CFA access [14].

The second method of registration is a prototypical, sonography-assisted application based on an ultrasound swipe of the femoral region and a deep-learning algorithm for registration. It also requires an optical tracking system, which tracks the needle as well as the sonography probe. The sonography-assisted registration is demonstrated in Supplementary Video S2 and has also been previously described [13].

For visualization, there were two available methods available.

**Figure 4:**
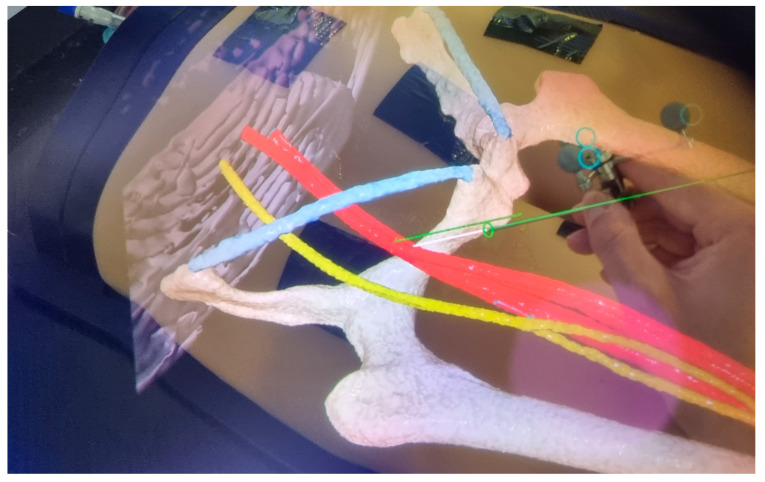
HMD visualization. The operators’ point of view is displayed. The green line represents the predefined trajectory aiming at the mid-level of the femoral head with a 0° coronal angle and a 45° sagittal angle.

The first method visualized the registration as well as the needle tracking in the field of view of the operator utilizing the HMD (HMD visualization) (Figure 4). It also included a visual representation of the predefined optimal trajectory of the needle. HMD visualization is presented in Supplementary Video S3.

**Figure 5:**
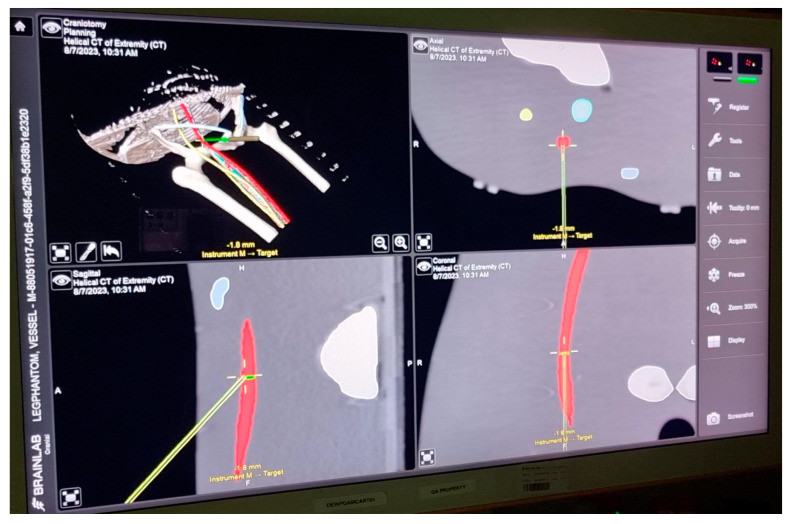
Monitor visualization. The needle is represented in the axial plane as well as with coronal and sagittal reconstructions. The green line represents the optimal trajectory for the operator to follow.

The second visualization method displayed the information on a conventional monitor in axial slices and two CT reconstructions (sagittal, coronal) as well as one three-dimensional reconstruction, also including needle tracking and planned trajectory (monitor visualization) (Figure 5). The monitor visualization is presented in Supplementary Video S4.

From the described methods of registration and visualization, the three navigated workflows were derived and compared to the control, as presented in Table 1.

**Figure 6:**
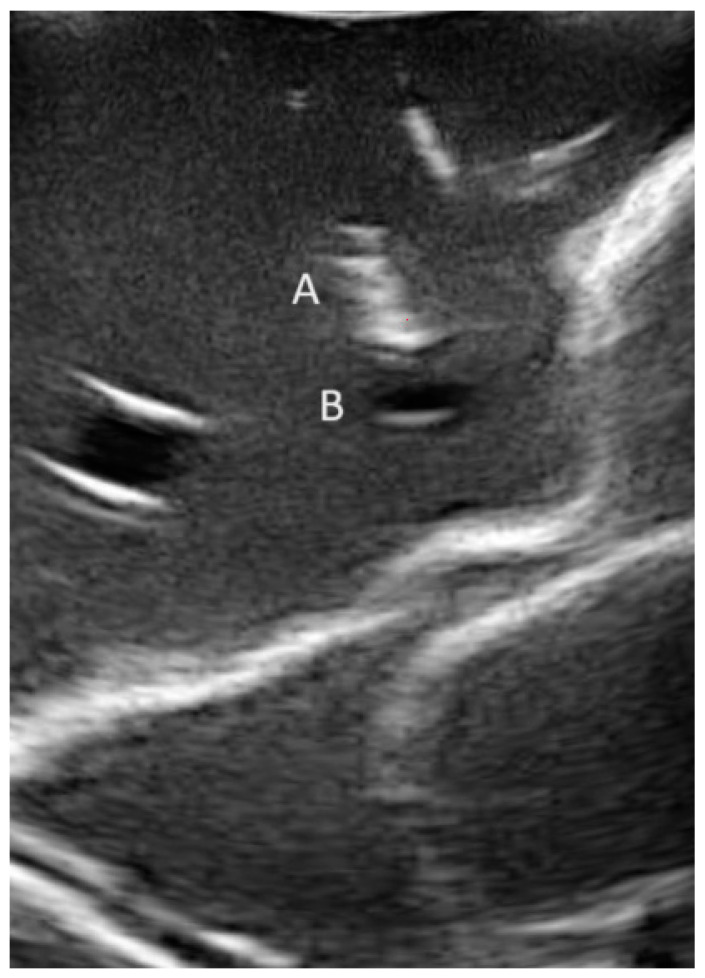
Conventional sonography-guided puncture in out-of-plane technique. A: needle; B: common femoral artery (CFA).

Overall, 160 CFA access procedures were performed: 80 procedures on the right side, and 80 procedures on the left side. Furthermore, 40 conventional sonography-guided punctures were compared with the three alternative workflows, each evenly compared with 40 attempts. The sonography-guided technique in the control group is illustrated in Figure 6. Punctures were performed in total by 10 operators, 5 of which were experienced (experts) in the technique of CFA access and 5 of which had no or minimal prior experience (non-experts). Experts were 1 senior vascular surgeon, 1 fellow vascular surgeon, 1 neurosurgeon, 1 radiologist, and 1 medical student with experience in CFA access from prior experiments. Non-experts were staff with no prior experience of practically performing needle interventions and limited prior experience with medical imaging. In summary, every operator performed two attempts per side and per method. Operators, sides, and methods were systematically arranged to ensure an even distribution and sequence of side, method, and experience throughout the experiment.

### 2.4. System Usability of Registration and Visualization Methods

For the usability assessment of the registration methods, each operator graded the paired-point registration as well as the sonography-assisted registration using the system usability scale (SUS). Additionally, the SUS was also used to grade the usability of HMD and monitor visualizations, respectively.

### 2.5. Definitions

#### 2.5.1. Technical Success

Technical success was defined as successfully inserting a 0.018” guidewire (Glidewire advantage, Terumo, Tokyo, Japan) and subsequently visualizing it within the vessel radiographically.

#### 2.5.2. Positional Errors

Positional errors were assessed by cone-beam computed tomography following each puncture (Cios Spin, Siemens Healthineers AG, Forchheim, Germany). The positional error was quantified in four defined measurements: distance of the needle entry point from the mid-level of the femoral head in centerline reconstruction, distance of the centerline to the needle tip in the axial plane, insertion angle in the coronal plane (difference of 0° with the planned trajectory), and insertion angle in the sagittal plane (difference of 45° with the planned trajectory).

### 2.6. Statistical Analysis

Technical success rates were compared among subgroups using the Chi^2^-test. For positional accuracy, mixed effects models were developed to account for different sides, operator experience levels, and workflows used. The models were developed for all 4 endpoints that measure positional accuracy: distance of the needle entry site to the mid-level of the femoral head, the distance of the needle tip to the centerline, and the insertion angle in the coronal plane as well as the sagittal plane. The mixed effects model included fixed effects for the side of the procedure (anatomical difficulty), the method deployed, and the operator experience. The random effect of operator was included to capture individual variability between operators. The right side, expert-level operators, and the conventional sonography-guided technique were used as reference categories.

Statistical analysis was performed using R Statistical Software (Version 3.4.1., R Foundation for Statistical Computing, Vienna, Austria) [31]. Statistical significance was defined as α < 0.05. The system usability score was calculated based on the system usability scale and normalized to 100 as the maximum score. This requires subtracting 1 point from each odd-numbered question, and each even-numbered question’s value was subtracted from 5. The score was added up and multiplied by 2.5. A score of 100 indicates high usability [32].

## 3. Results

### 3.1. Technical Success

Overall, technical success was achieved in 144/158 (91.1%) attempts. Two attempts were excluded from the analysis. In one excluded attempt, the Magic Leap 2 had a technical failure during the attempt, and in the other excluded attempt, the cone-beam CT that was used to estimate positional error had insufficient quality, leaving 158 punctures for analysis. The technical success rate in the expert group was significantly higher with 78/79 (98.7%) when compared with the non-expert group (66/79 (83.5%), *p* = 0.002). With regard to the method used, the control group had a similar success rate with 20/20 (100%) in the expert group and 19/20 (95.0%) in the non-expert group (*p* = 1.0). The navigated techniques had a success rate of 58/59 (98.3%) in the expert group and 47/59 (79.7%) in the non-expert group, which was a statistically significant difference (*p* = 0.003). Technical success is displayed in Table 2.

### 3.2. Endpoints (1–4) in the Mixed Effects Model

#### 3.2.1. Distance from the Needle Entry Site to the Mid-Level of the Femoral Head

A left-sided procedure did not significantly influence the distance from the targeted level relative to the femoral head (estimate: 0.98, CI: [−2.24]–[4.20], *p* = 0.550). Compared with the reference category, workflow 2 and workflow 3 were associated with a significant decrease in the distance of needle entry to the mid-level of the femoral head (workflow 2: estimate: −8.3, CI: [−12.83]–[−3.78], *p* < 0.001; workflow 3: estimate: −8.52, CI: [−13.1]–[−3.93], *p* < 0.001). Non-expert operators were associated with a significant increase in the distance of needle entry to the targeted plane (estimate: 4.43, CI: [0.19]–[8.66], *p* = 0.040).

#### 3.2.2. Distance of the Centerline to the Needle Tip in the Axial Plane

Neither the side, the operator experience, nor the method used had a significant effect.

#### 3.2.3. Insertion Angle in Coronal Plane

While left-sided procedures as well as experience levels did not have a significant effect, the methods used had a significant effect. While workflow 1 and 3 were associated with a slight increase in coronal insertion angulation when compared to the reference category (workflow 1: estimate: 2–56°, CI: [0.46]–[4.66], *p* = 0.017; workflow 3: estimate: 2.58°, CI: [0.45]–[4.71], *p* = 0.018), workflow 2 was associated with a decrease in angulation (estimate: −2.65°, CI: [−4.75]–[−0.56], *p* = 0.014).

#### 3.2.4. Insertion Angle in Sagittal Plane

Left-sided puncture and navigated workflows each had significant effects with a lower sagittal insertion angle (side: estimate: −0.92°, CI: [−1.84]–[−0.01], *p* = 0.047; workflow 1: estimate: −1.52°, CI: [−2.80]–[−0.24], *p* = 0.021; workflow 2: estimate: −1.59°, CI: [−2.87]–[−0.30], *p* = 0.016; and workflow 3: estimate: −1.44°, CI: [−2.74]–[−0.13], *p* = 0.031).

**Figure 7:**
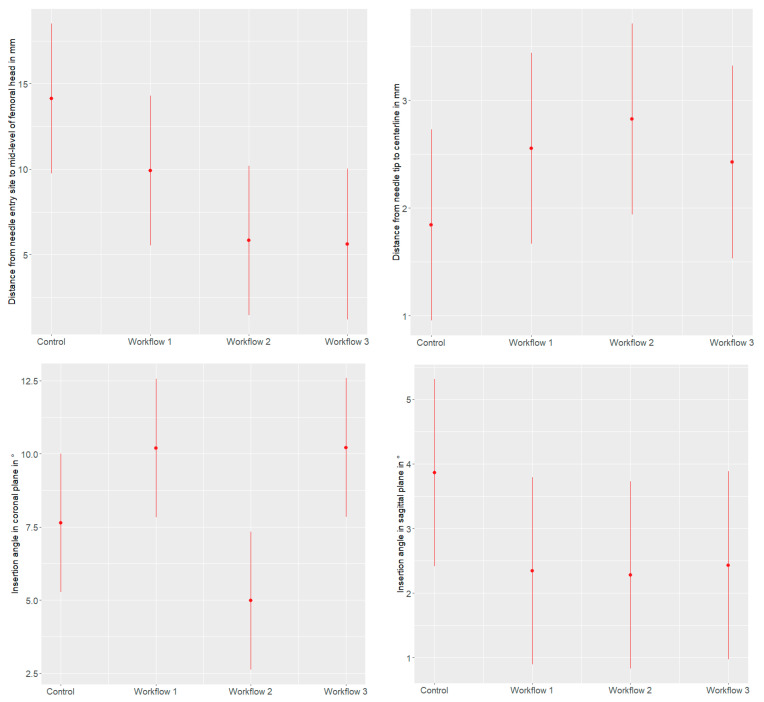
Effect plots demonstrating the effect of the different workflows on the distance from the needle entry site to the mid-level of the femoral head (in mm), the distance from the needle tip to the centerline (in mm), the insertion angle in the coronal plane (in °), and the insertion angle in the sagittal plane (in °), with 95% confidence intervals.

The mixed effects model results are presented in Table 3 and Figure 7.

### 3.3. System Usability

#### 3.3.1. Registration

The usability of paired-point registration was scored at a mean of 75.5 (+/−20.6) points, while usability of the sonography-assisted technique was rated at 61.5 (+/−24.1) points.

#### 3.3.2. Visualization

The usability of conventional monitor visualization was scored at 76.8 (+/−13.1) points. The usability of HMD visualization received 63.8 (+/−21.7) points.

## 4. Discussion

MxR has previously been described as a promising technology that could improve the accuracy and safety of navigational tasks in the future, such as common femoral arterial access and others [12,33]. Accurate registration of the physical and virtual patients is critical for these sorts of applications, while at the same time, is maintaining the clinical feasibility of the workflow. Soft tissue such as vasculature or organs are especially difficult to register, due to potential deformations and movements of the targeted structures during the performance of the said tasks either by the introduction of materials, the compression of surrounding tissue, or patient’s movements during respiration or muscle activity.

In this study, we investigated three different workflows for navigated common femoral access on a realistic phantom model and compared it with a conventional sonography-guided technique as a control. Furthermore, we aimed to introduce different levels of anatomical difficulty and operator experience to the analysis.

The technical success rates, defined as the successful introduction of a guidewire within the vessel, were high for the control group of experts (100%) and non-experts (95%) alike, with no statistically significant differences. This demonstrates that sonography guidance is per se an effective method to achieve successful guidewire introduction, especially in a model with a rather straightforward anatomy, with good sonographic visualization of the target, and no calcifications or excessive adipose tissue.

Interestingly, when comparing technical success rates in the navigated methods, the non-experts performed significantly worse than the experts. This might be due to inexperience with regard to haptic feedback when introducing a guidewire. While experts can react to slightly increased resistances with subtle directional corrections, operators with no or minimal prior experience tend to advance the wire regardless. Unfortunately, in the present experiment, the virtual representation of the needle as well as the target did not allow to view the needle within the target vessel, which added difficulty. In future experiments, the virtual target vessel should be displayed more transparently to enable the operator to see the virtual representation of the needle inside the target. When using sonography, even a novice can easily visualize the needle tip within the lumen in this experimental set-up. Additionally, for non-experts, handling additional equipment and steps in the workflow such as markers and the head-mounted display might be more challenging. Moreover, comprehending the two-dimensional monitor visualization in the navigated workflow 3 can be difficult for inexperienced observers who are not accustomed to interpreting computed tomography imaging let alone its reconstructions in multiple planes simultaneously.

While sonography was demonstrated as an effective method for experts and non-experts to advance a guidewire, in clinical practice, mere puncture and introduction of a guidewire is not a suitable indicator of a successful access procedure. Besides guidewire introduction, the precise needle entry location on the anterior wall of the common femoral artery is equally important to prevent complications [22]. Mixed effect model analysis in this study revealed that the distance from the needle entry site to the targeted entry site is significantly shorter for navigated techniques than for the control group, for experts as well as non-experts. This highlights that three-dimensional visualization and visual presentation of a planned trajectory could increase the safety of CFA access, by lowering the risk of accidental profound or superficial femoral artery puncture, puncture through the inguinal ligament, or even puncture above the inguinal ligament, each of which could lead to complications [15,22,23,24,25,26,27]. Therefore, this analysis generally supports the hypothesis that navigating CFA access could increase the overall procedural safety [34]. Additionally, in the mixed effect model, the insertion angles in coronal and sagittal planes were significantly different in the navigated workflows from the control, although the difference was very limited and of questionable clinical significance.

With regard to the method of registration, it could be demonstrated that the prototypical sonography-assisted registration software was as accurate for the performance of CFA access as the more widely adopted paired-point registration. While paired-point registration has been validated in human use, the sonography-assisted registration method still lacks investigation in terms of its safety and effectiveness in patients and is therefore a subject for future studies. Although, as shown in usability grading, the prototype requires further reduction in of complexity to increase its practical usability, and sonography-assisted registration in principle appears more usable in future routine work from a clinical perspective. It eliminates the need of fiducial markers and allows for easily repeatable registration [13].

For the purposes of visualization, improved results in the non-expert group with the HMD indicate that three-dimensional visualization might be more intuitive than reconstructed computed tomography images. However, for experts, this advantage might be of limited relevance.

For all navigated workflows, cost as well as infrastructural requirements were high. All navigated workflows presented herein are in their current form not justified for evaluation in clinical use for CFA access due to their complexity and cost. However, CFA access might be a gate opener to develop the necessary prerequisites for more advanced, navigated vascular surgical and interventional procedures in the future. Furthermore, the technique might assist during the training of CFA access and provide a better understanding of the two-dimensional images acquired during conventional sonography-guidance by visualizing the underlying three-dimensional anatomy for the trainee.

### Limitations

This study exhibits several limitations. While this is one of the largest studies investigating MxR for navigational tasks in vascular surgery, it is still in a phantom model experimental stage with a limited number of operators and attempts. Results are not generalizable to its use in real patients. Although we aimed to minimize learning curve effects by expanding the number of observers and limiting the number of attempts per side and method, and we arranged the methods so that the sequence of methods, operators, and sides were evenly distributed, learning effects might still bias results. Furthermore, we could only differentiate two levels of anatomical difficulty with regard to the depth and diameters of the targeted vessels, but we were not able to simulate different levels of obesity or vessel calcification. This also limited our ability to investigate the performance of registration methods with varying deformation of the soft tissue due to pressure exerted via the ultrasound probe. Future experiments need to include a broader variety of anatomy. Additionally, the system usability scaling was not performed for the control of conventional sonography, which did not allow for the comparative assessment of the usability of the navigated workflows with the control group. Furthermore, there was no timing provided for each individual attempt. In its current version, all navigated techniques required preprocessing and were associated with an increase in the overall required time compared to sonography. This time was not measured in a standardized manner for this study, but generally ranged between 1 and 3 min.

## 5. Conclusions

According to this phantom model experiment, navigated techniques appear as a potentially beneficial adjunct for safe CFA access in the future. Continued developments should aim to reduce workflow complexity, avoid advanced optical tracking systems, and offer alternative methods of registration. Furthermore, supplementing the conventional sonographic visualization for CFA access by three-dimensional registration could assist in resident training and safe access procedures in more complex cases in the future.

## Figures and Tables

**Table 1:** Registration and visualization in the control group as well as in workflow 1–3.

	Registration	Visualization
Control	Conventional sonography	
Workflow 1	Sonography-assisted (prototype) ^1^	HMD ^3^
Workflow 2	Paired point ^2^	HMD ^3^
Workflow 3	Sonography-assisted (prototype) ^1^	Monitor ^4^

^1^ The sonography-assisted registration is demonstrated in Supplementary Video S2. ^2^ Paired-point registration is demonstrated in Supplementary Video S1. ^3^ HMD visualization is demonstrated in Supplementary Video S3. ^4^ Monitor visualization is demonstrated in Supplementary Video S4.

**Table 2:** Technical success of CFA access.

	Expert	Non-Expert	*p*-Value ^1^
Technical success (N = 158) [n/N (%)] Overall	78/79 (98.7%)	66/79 (83.5%)	
	Right: 39/39 (100%)	Right: 35/39 (89.7%)	0.002
	Left: 39/40 (97.5%)	Left: 31/40 (77.5%)	
Control	20/20 (100%)	19/20 (95.5%)	
	Right: 10/10	Right: 10/10 (100%)	1.0
	Left: 10/10	Left: 9/10 (90%)	
Navigated	58/59 (98.3%)	47/59 (79.7%)	
	Right: 29/29 (100%)	Right: 25/29 (86.2%)	0.003
	Left: 29/30 (96.7%)	Left: 22/30 (73.3%)	
Workflow 1	20/20 (100%)	15/20 (75%)	
	Right: 10/10 (100%)	Right: 9/10 (90%)	
	Left: 10/10 (100%)	Left: 6/10 (60%)	
Workflow 2	20/20 (100%)	16/20 (80%)	
	Right: 10/10 (100%)	Right: 8/10 (80%)	
	Left: 10/10 (100%)	Left: 8/10 (80%)	
Workflow 3	18/19 (94.7%)	16/19 (84.2%)	
	Right: 9/9 (100%)	Right: 8/9 (88.9%)	
	Left: 9/10 (90%)	Left: 8/10 (80%)	

^1^ Chi^2^-test.

**Table 3:** Results of the mixed effects model.

	Distance from the Needle Entry Site to the Mid-Level of the Femoral Head (mm)			Distance of the Centerline to the Needle Tip in the Axial Plane (mm)		
Predictors	Estimates	CI	*p*	Estimates	CI	*p*
Intercept	13.15	[8.8]–[17.5]	<0.001	1.85	[1.0]–[2.7]	<0.001
Left side	0.98	[−2.2]–[4.2]	0.550	−0.01	[−0.7]–[0.7]	0.976
Workflow 1	−4.22	[−8.7]–[0.3]	0.068	0.71	[−0.3]–[1.7]	0.171
Workflow 2	−8.30	[−12.8]–[−3.8]	<0.001	0.98	[−0.04]–[2.0]	0.059
Workflow 3	−8.52	[−13.1]–[−3.9]	<0.001	0.59	[−0.5]–[1.6]	0.265
Non-expert operator	4.43	[0.2]–[8.7]	0.040	0.63	[−0.1]–[1.4]	0.091
Random Effects						
σ^2^	104.89			5.32		
τ_00operateur_	4.82			0.00		
ICC	0.04					
N_operateur_	10			10		
Observations	158			158		
Marginal R^2^/Conditional R^2^	0.137/0.175			0.041/NA		
	Insertion angle in coronal plane (°)			Insertion angle in sagittal plane (°)		
Predictors	Estimates	CI	*p*	Estimates	CI	*p*
Intercept	7.01	[4.7]–[9.4]	<0.001	4.79	[3.3]–[6.2]	<0.001
Left side	0.62	[−0.9]–[2.1]	0.413	−0.92	[−1.8]–[−0.01]	0.047
Workflow 1	2.56	[0.5]–[4.7]	0.017	−1.52	[−2.8]–[−0.2]	0.021
Workflow 2	−2.65	[−4.8]–[−0.6]	0.014	−1.59	[−2.9]–[−0.3]	0.016
Workflow 3	2.58	[0.5]–[4.7]	0.018	−1.44	[−2.7]–[−0.1]	0.031
Non-expert operator	0.51	[−2.1]–[3.1]	0.700	−0.24	[−1.8]–[1.4]	0.767
Random Effects						
σ^2^	22.56			8.45		
τ_00operateur_	2.90			1.10		
ICC	0.11			0.11		
N_operateur_	10			10		
Observations	158			158		
Marginal R^2^/Conditional R^2^	0.161/0.257			0.066/0.173		

## Data Availability

Data are available upon reasonable request from the corresponding author.

## Supplementary Materials

The following supporting information can be downloaded at: https://www.mdpi.com/article/10.3390/jimaging10040076/s1.

## Abbreviations

MxR	Mixed reality
HMD	Head-mounted display
CFA	Common femoral artery
CTA	Computed tomography angiography
SUS	System usability scale
CI	Confidence interval
